# Management of the soybean cyst nematode *Heterodera glycines* with combinations of different rhizobacterial strains on soybean

**DOI:** 10.1371/journal.pone.0182654

**Published:** 2017-08-03

**Authors:** Yuanyuan Zhou, Yuanyuan Wang, Xiaofeng Zhu, Rui Liu, Peng Xiang, Jingsheng Chen, Xiaoyu Liu, Yuxi Duan, Lijie Chen

**Affiliations:** 1 Nematology Institute of Northern China, Shenyang Agricultural University, Shenyang, Liaoning, China; 2 Heihe Branch, Heilongjiang Academy of Agricultural Sciences, Heihe, Heilongjiang, China; 3 Daqing Branch, Heilongjiang Academy of Agricultural Sciences, Daqing, Heilongjiang, China; Henan Agricultural University, CHINA

## Abstract

Soybean cyst nematode (SCN) is the most damaging soybean pest worldwide. To improve soybean resistance to SCN, we employed a soybean seed-coating strategy through combination of three rhizobacterial strains, including *Bacillus simple*, *B*. *megaterium* and *Sinarhizobium fredii* at various ratios. We found seed coating by such rhizobacterial strains at a ratio of 3:1:1 (thereafter called SN101) produced the highest germination rate and the mortality of J2 of nematodes. Then, the role of soybean seed coating by SN101 in nematode control was evaluated under both greenhouse and two field conditions in Northeast China in 2013 and 2014. Our results showed that SN101 treatment greatly reduced SCN reproduction and significantly promoted plant growth and yield production in both greenhouse and field trials, suggesting that SN101 is a promising seed-coating agent that may be used as an alternative bio-nematicide for controlling SCN in soybean fields. Our findings also demonstrate that combination of multiple rhizobacterial strains needs to be considered in the seed coating for better management of plant nematodes.

## Introduction

The soybean cyst nematode (SCN *Heterodera glycines*) is one of the most economically important pest on soybean (Glycine max) worldwide. Average yield loss of soybean due to SCN infection is up to 30% in China, even up to 70% in some fields of Northeast China[1]. SCN is also continuous yield limitation in USA [2–4]. Infection of SCN leads to formation of a syncytial feeding site in soybean, which functions as metabolic sinks to nourish the nematodes [5]. As a result of nematode infection, the nodulation and nitrogen fixation is suppressed in soybean, thereby resulting in plant nutrient deficiency symptoms [6].

Various practices including crop rotation, resistance cultivars and nematicide application have been used to control SCN. However, SCN-resistant varieties have been limited in practice by long term breeding [7], various photoperiods in different areas and genetic diversity of SCN races in the fields [8]. Further, many nematicides have already been removed from the market because of their negative effects on public and environmental health [9]. However, seed coating approach, which can minimize pesticide dust-off [10], is effective to control plant pests and diseases [11]. Seed coating on soybean using insecticide chitosan improves seed germination rate, root establishment and yields [12,13]. Therefore, developing alternative safe and efficient way such as seed coating is urgent for SCN control.

Bacteria are the most important biocontrol agents. Individual rhizosphere and/or endophytic bacteria, such as *Rhizobium* spp., *Bacillus firmus* strain GB126 and *B*. *subtilis* could display biological activity against nematodes [14–18]. Co-application of *Paecilomyces lilacinus* and *B*. *firmus* provides effective control of root-knot nematodes in the pot experiment [19]. In contrast, it is not clear whether the combination of several beneficial bacteria plays a synergistic effect to control SCN in the greenhouse or in the field, although there are potential of synergic effect.

Our previous studies showed that individuals of three strains, namely *Sinarhizobium fredii* strain Sneb183 [20], *B*. *megaterium* strain Sneb 482 [21] and *B*. *simplex* strain Sneb545 [22], reduced the activity of SCN in the lab condition. This study was aimed to evaluate the effect of mixture of the three bacterial strains as a seed-coat treatment of soybean on SCN control and soybean yield under greenhouse and field conditions.

## Materials and methods

### Nematode inoculum

For the greenhouse experiment, SCN race 1 was usedThey were collected from the naturally infested soil in Kangping County in Liaoning Province and identified using the method described by Riggs [23]. SCN second-stage juveniles (J2) were obtained by first collecting cysts on a set of sieves of 420 μm and 250 μm, surface-disinfecting with 0.5% NaOCl for 3 min, and washing three times with sterile water before placing in an incubator at 28°C to allow the J2 to hatch. The nematode inoculum was used for pot experiments. For the field experiment, soybean cyst nematode race 1 and 3 as identified previously [23] were naturally infested in the field of Kangping and Daqing, respectively.

### Preparation of bacterial strains and biocontrol agents

*S*. *fredii* strain Sneb183, used in this study was originally isolated from the rhizosphere of pines in Liaoning province, China [20]. *B*. *megaterium* strain Sneb482 was isolated from soybean root nodule and *B*. *simplex* strain Sneb545 from the soil around rhizosphere of soybean in Liaoning province, China [22]. Sneb183 was grown on yeast morphology agar (YMA) medium, Sneb482 and Sneb545 on beef extract peptone (NA) medium respectively, for 48h at 28°C in the dark. A single colony was selected and inoculated in 100 mL tryptone yeast (TY) for Sneb183 or NA liquid medium for Sneb482 and Sneb545 in a flask, which was then incubated for 48 h at 28°C on a rotary shaker at 150 rpm. Then the bacteria density was adjusted to a final optical density (OD) of 2 at 560 nm for Sneb183, 0.3 at 580nm for Sneb545 and 0.25 for Sneb482, containing approximately 1.0×10^9^ CFU ml^-1^, with the growth liquid medium and stored at 4°C for use.

### Plant material and soil

In germination and greenhouse experiments, the soybean cultivars Liaodou15 (provided by the Liaoning Academy of Agricultural Sciences) and Hefeng50 (provided by the Heilongjiang Academy of Agricultural Sciences) were used, which are susceptible to SCN. The seeds were surface-sterilized with 75% ethanol for 1 min, followed by 1.5% NaOCL for 3 min, and then rinsed with sterile water multiple times and air-dried. The sterilized seeds were then coated with different hundred biocontrol agents at a ratio of seed weight to bacteria suspension mixture volume at 1:70 and air-dried. Field experiments were conducted in two locations for 2 years from 2013 to 2014. Liaodou15 was used in the field of Kangping County, Liaoning Province and Hefeng50 in Daqing city, Heilongjiang Province.

For the pot experiment in the greenhouse, soil material bought from Shenyang Jintian Horticulture Research Institute wwere autoclaved at 137.9 kPa for 20 min. Then 500 g of mixed soil was put into 11-cm-diam plastic pot. In the field, the native soil contained organic substrate 9.20 (g/kg) in Kangping and 5.39 (g/kg) in Daqing. And the available nitrogen, phosphorus and potassium (mg/kg) in the two fields were 116.14, 9.79, and 111.11 in Kangping and 44.76, 17.51 and 94.75 in Daqing, respectively.

### In vitro Effect of the three bacteria and the combination on nematode mortality

Nematicidal effect of *S*. *fredii*, *B*. *megaterium* and *B*.*simplex* was evaluated against SCN under laboratory conditions. One hundred of SCN juveniles were transferred to the suspensions of microorganisms (10^9^ CFU ml^-1^ of *S*. *fredii*, 10^9^ CFU ml^-1^ of *B*. *megaterium* and 10^9^ CFU ml^-1^ of *B*. *simplex*) separately or combined in different proportions in sterilized Petri dishes, and separated sets of Petri dishes were maintained for 48h for observation. Five replicates were conducted for each treatment. Then, mortality of nematodes was assessed and confirmed by touching the juvenile with fine needle. Five treatments (SN101 1–5) were used for the assay.SN101-1 contained *B*. *simple*, *B*. *megaterium* and *S*. *fredii* with 3:1:1, and SN101-2 contained them with 1:1:1, SN101-3 contained only *B*. *simple*, and SN101-4 contained only *B*. *megaterium*, SN101-5 contained only *S*. *fredii* and sterilized distilled water treatment was used as blank control. Five replicates were conducted for each treatment and the experiment was repeated.

### Effect of different bioagents on the seedling growth of soybean

In order to select the best ratio of bacterial mixture for seed-coating, an initial germination test was conducted. Early experiment proved increase of germination when different strains were applied together. Seeds were sterilized as described above. The sterilized seeds were then coated with SN101-1, SN101-2, SN101-3, SN101-4 and sterile water, then air-dried again. The coated seeds were placed on wet blotters and germination test was conducted by using the filter paper method [24]. They were incubated at 28 ± l°C for one week. The filter papers were moistened with sterile water as needed and the seeds germination was recorded daily. Seedling vigor was determined by its vigor index (VI), using the following formula described by Baki and Anderson [25] for calculation.

Vigor index (VI) = (mean shoot length+mean root length) × percentage of germination

The optimal ratio of the bacterial mixture treatment was determined based on the seedling vigor value, and referred as SN101.

### Effect of SN101 bioagent on SCN infection under greenhouse condition

To further verify the effect of SN101, a commercial seed coating agent (BFA) was selected, and a pot experiment under greenhouse was designed. Soil and seeds were treated as described above. Seedlings of soybean (Liaodou 15) were raised in 11-cm-diam plastic pots each containing 500g sterilized soil. The SN101-coated soybean seeds were sown in these pots and maintained at 27± 3°C under a 16 h/8 h light/dark cycle. Uncoated seeds were sown as a control (denoted as CK), and seeds coated with a commercial biological seed coating agent, named BFA which contained 40% of bio-control bacteria (Manufacturer: Zhong Cai Sheng Shi biotechnology development co., Inner Mongolia MoQi branch) were sown as a bio-nematicide control. There are ten pots per line, and one seed in a pot, five lines were conducted for each treatment.

The soybean seedlings were inoculated with 3000 second-stage juveniles (J2) per plant at their two-leave stages. The juveniles were dispensed in 8 ml of water and distributed evenly into the soil around the seedling. Pots were arranged in randomized block design on a bench in the greenhouse at 25±2°C. The plants were taken out from the pots 30 days after inoculation, washed and blotted dry. The measured data included plant height, root length, cysts/100 cm^3^ mixed soil. The soybean roots were stained with 0.1% acid fuchsine [26,27] and the SCN juveniles from J2 to J4 stages inside the roots were counted. Six replicates were conducted for each treatment and the experiment was repeated.

### Effect of SN101 bioagent on SCN infection under field conditions

Field trials were conducted in the soybean fields naturally infested by SCN at Kangping County in Liaoning Province and Daqing city in Heilongjiang Province in 2013 and 2014. In 2013, the seeding date was May 12 and May 20, and harvested in the Oct. 17 and Oct. 10 in Kangping and Daqing, respectively. In 2014, the seeding and harvesting date was May 8 and Oct. 8, separately, in Kangping and May 18 and Sep. 30 in Daqing. As the greenhouse trial, the optimized seed coating formula SN101 was used for evaluation. Also, uncoated seeds (denoted as CK) and seeds coated with BFA (a commercially bio-nematicide) were sown as two controls. Each experimental unit consisted of a five-row plot (5×5m) planted with 100 soybean seeds per row. For each treatment there are five unions. The field trials were arranged as a randomized complete block design with five replicates for each treatment each year.

Twenty-five soybean seedlings were randomly selected from each replicate (using Z-shaped method) after 30–35 days seeding, respectively. The following data were recorded: plant height, root length, above-ground and under-ground fresh weight (data not shown), the number of cysts on the roots and in the soil around the roots, the number of eggs per cyst, and the number of SCN juveniles (J2, J3, J4) inside the roots. The roots were carefully removed from soil and cysts were counted directly, and then the plants were carefully taken back to laboratory. The fresh weight of the root and shoot for each plant was weighed by electronic analytical balance immediately, then the height of the shoot and root was measured by a plastic ruler. Nematodes inside the roots were stained as described previously [26] and the different stages were determined and counted under the microscope. For soybean yield estimation, twenty-five soybean plants were randomly collected as above and surveyed in each unit at harvest. The plant heights, pod numbers per plant, seed numbers per plant, and 100-seed weight were measured. The field tests were repeated 2 years from 2013 to 2014, and the average number was calculated to evaluate the effect of SN101 on SCN control.

We also conducted the field experiments in scientific experimental stations of Heihe and Jiusan for from 2012 to 2016 and measured the plant yield at harvest. In Heihe station, the soybean cultivar Heihe43 was used, and in Jiusan station the cultivar Kenjiandou28 was used. There are only one plot with the area of 416 m^2^ for each treatment each year.

### Statistical analysis

The growth indices of soybean growth, soybean yield, cyst number, egg number per cyst, and juvenile number per root were used in statistical analysis. Analysis of variance (ANOVA) was used individually for each field experiment, and the significance of differences among the treatments was determined according to Least Significant Difference (LSD) (*P* ≤ 0.05). To analyze the effect of SN101 seed coating on SCN infection, soybean growth and soybean yield under field condition, a multiple-factor ANOVA was performed. The factors included years (two levels), sites (two levels) and treatment (three levels). When an overall ANOVA indicated significant effects of the factors or their interactions, the means were compared. Data were analyzed using the software SPSS 17.0.

## Results

### In vitro assay

The mortality of SCN J2 was significantly increased in all treatments compared to the blank control at 12h and 48 h. The combination treatment SN101-1 resulted in highest mortality rate at 24 h (91%) and 48 h (up to 97%). The mortality rates with other four SN101 treatments could reach 77–84% at 24 h and 84–92% at 48 h, only 4–6% for control treatment (Table 1).

**Table 1:** Effect of five SN101 biocontrol agents on mortality of second-stage juveniles of *Heterodera glycines*.

Treatments	Mortality rate (%)	
	After 24 h	After 48 h
SN101-1	91.2±1.5a	97±0.84a
SN101-2	83.4±1.33b	91.6±0.87b
SN101-3 (*B*. *simple*)	81.2±1.77bc	90.6±1.36b
SN101-4 (*B*. *megaterium*)	84±0.55b	89.4±1.36b
SN101-5 (*S*. *fredii*)	77.4±2.29c	84.2±1.43c
Control	4±1.22d	6±0.71d

Mortality was determined by checking mortility after 24h and 48h. The data in the table are mean ± SE and means on the same column followed by different letters indicate significant differences based on a LSD test (*P* ≤ 0.05, n = 100). SN101-1 contained *B*. *simple*, *B*. *megaterium* and *S*. *fredii* with 3:1:1, and SN101-2 contained them with 1:1:1, the concentration of each strain was 1×10^9^ cfu/ml, SN101-3 contained only 1×10^9^ cfu/ml of *B*. *simple*, and SN101-4 contained only 1×10^9^cfu/ml of *B*. *megaterium*, SN101-5 contained only *S*. *fredi*i and sterilized distilled water treatment used as blank control.

### Effect of SN101 bioagents on soybean seed germination and seedling vigor

All seed coating agents increased the percentage of seed germination and there are no significant difference among them (Table 2). The highest VI was recorded in seeds coated with SN101-1 and SN101-4. Moreover, SN101-1 seed coating has the most beneficial effect on the shoot length and root length when compared to other treatments and control (*P* ≤ 0.05). Therefore, SN101-1 seed coating (thereafter called SN101) was used in the following greenhouse and field experiments.

**Table 2:** Effect of biocontrol agents on soybean seed germination and seedling vigor (*P* ≤ 0.05).

Treatments	Germination (%)	Mean Shoot Length (cm)	Mean Root Length (cm)	Vigor index(VI)
SN101-1	94.33±1.2a	6.49±0.19a	11.69±0.32a	1714.92±42.68a
SN101-2	94±3.05a	5.28±0.15bc	9.25±0.32b	1365.82±38.82b
SN101-3 (*B*. *simple*)	94.67±1.76a	5.18±0.13c	9.50±0.31b	1389.76±34.71b
SN101-4 (*B*. *megaterium*)	94.67±2.73a	6.11±0.22ab	10.96±0.38a	1616.02±49.14a
Uncoated	87±1.0b	6.06±0.54ab	9.44±0.35b	1348.5±61.91b

The data in the table are mean ± SE and means on the same column followed by different letters indicate significant differences based on a LSD test (*P* ≤ 0.05, n = 200). SN101-1 contained *B*. *simple*, *B*. *megaterium* and *Sinarhizobium fredii* with 3:1:1, and SN101-2 contained them with 1:1:1, the concentration of each strain was 1×10^9^ cfu/ml, SN101-3 contained only 1×10^9^ cfu/ml of *B*. *simple*, and SN101-4 contained only 1×10^9^cfu/ml of *B*. *megaterium*, sterile water treatment used as blank control for seed coating.

### Effect of SN101 on SCN population and soybean growth under greenhouse conditions

**Fig 1:**
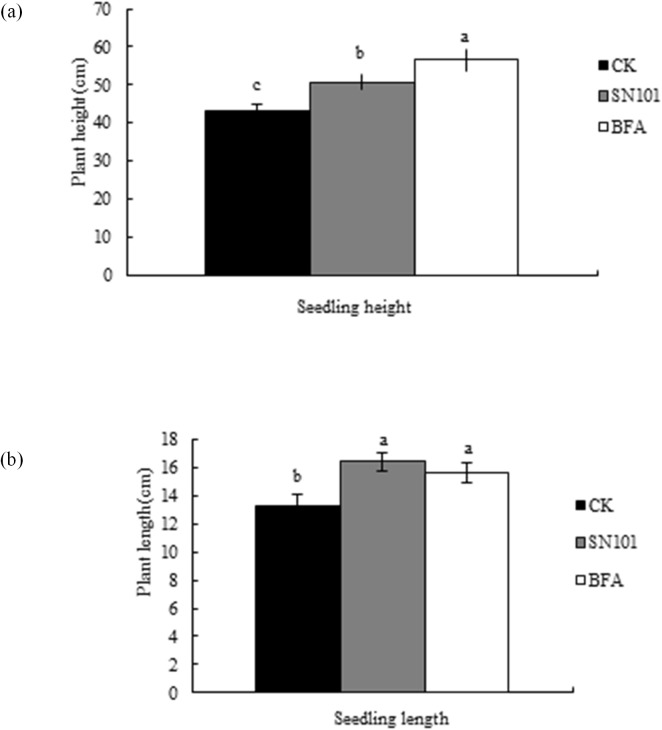
Effects of SN101 seed coating on the control of SCN infestation in soybean under greenhouse condition. Plants were treated with biocontrol seed coating-SN101, chemical seed coating-BFA and uncoated-CK. The number of juveniles and cysts was measured after nematode inoculation 30 days. The data in the figure are mean ± SE and means on the same column followed by different letters indicate significant differences based on a LSD test (*P* ≤ 0.05, n = 12).

**Fig 2:**
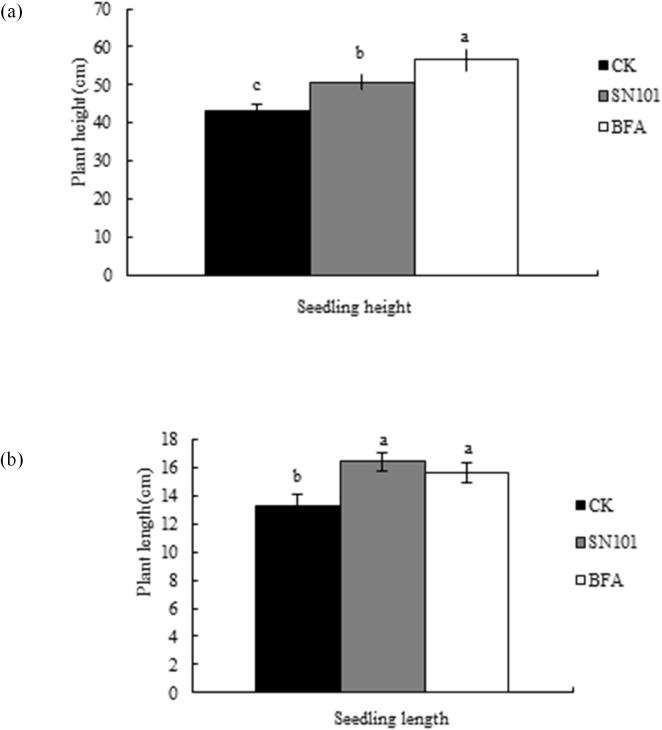
Effects of SN101 seed coating on soybean height and root length under greenhouse condition. Plants were treated with biocontrol seed coating-sn101, chemical seed coating-BFA and uncoated-CK. The plant height and root length was measured after nematode inoculation 30 days. The data in the figure are mean ± SE and means on the same column followed by different letters indicate significant differences based on a LSD test (*P* ≤ 0.05, n = 125).

At 30 days post inoculation, cyst numbers in the soil per pot (Fig 1A) and SCN juveniles inside the roots were reduced by 18.13% and 26.72% (*P* ≤ 0.05), respectively, after seeds were treated with SN101, and reduced by 6.79% and 23.64% (*P* ≤ 0.05) in BFA treatment, when compared to uncoated ones. Meanwhile, the seedling height and root length of the soybean plants were increased by 17.12% and 43.55% (*P* ≤ 0.05), respectively, after treatment with SN101, compared to the uncoated control (Fig 2).

### Application of SN101 seed coating for nematode control under field conditions

Multiple factor ANOVA analysis indicated that years, sites and treatments had a significant effect on the nematode dynamics except for the effect of sites on the number of eggs per cyst. But there are limited interactions among the factors: years, sites, and treatments, except for the effect of significant interactions on the number of cysts per root system (Table 3).

**Table 3:** Analysis of variance of three factors (years, sites and treatments).

Index	Y (Year)	S (Sites)	T (Treatments)	Y*S	Y*T	S*T	Y*S*T
The number of cysts per root system	6.97*	769.31*	73.16*	48.89*	15.42*	8.70*	48.41*
The number of cysts per 100g rhizosphere soil	4.2*	9.74*	38.58*	16.96*	2.249ns	1.41ns	0.28ns
The number of juveniles per gram of root	168.67*	62.32*	8.14*	8.30*	0.07ns	1.65ns	0.03ns
The number of eggs per cyst	13.13*	2.52ns	73.35*	2.29ns	2.70ns	4.08*	0.10ns
Seedling plant height	859.49*	64.99*	0.82ns	59.07*	3.59*	5.92*	0.61ns
Mature plant height	164.76*	1340.01*	74.82*	76.49*	9.07*	1.52ns	5.35*
Seedling root length	496.53*	0.002ns	4.27*	0.22ns	3.06*	0.43ns	0.89ns
Pods number per plant	56.40*	132.52*	38.04*	201.29*	6.18*	4.21*	11.44*
Seeds number per plant	13.86*	6.77*	25.03*	233.57*	2.70ns	2.07ns	1.48ns
100-seed weight (g)	244.51*	807.71*	3.85*	15.13*	0.42ns	0.90	1.2

ns, non-significant at *P* ≤ 0.05 level

* *P*≤ 0.05 levels. *Y*, year; *S*, sites; *T*, treatments; Y*S, year and sites interaction; Y*T, year and treatments interaction; S*T, sites and treatments; Y*S*T, year and sites and treatments interaction.

**Fig 3:**
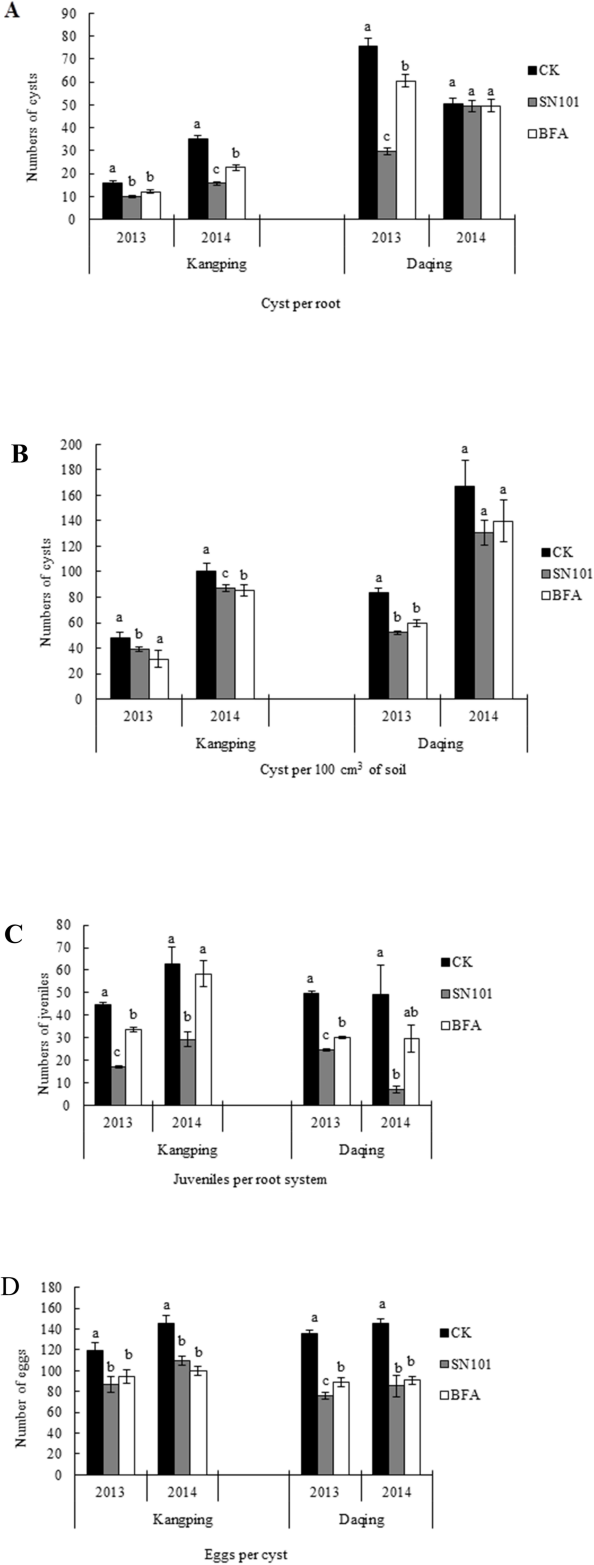
SN101 seed coating inhibited SCN infection under soybean filed conditions in 2013 and 2014. Plants were treated with biocontrol seed coating-SN101, chemical seed coating-BFA and uncoated-CK. The number of juveniles in roots, cysts in soil and in roots, and eggs per cyst was measured after planted 30–35 days. The data in the figure are mean ± SE and means on the same column followed by different letters indicate significant differences based on a LSD test (*P* ≤ 0.05). **A.** the number of cysts per root system (n = 125), **B.** the number of cysts per 100 g of rhizosphere soil (n = 25), **C.** the number of juveniles per gram of root (n = 125), **D.** the number of eggs per cyst (n = 25).

We found that the cyst number per root system (Fig 3A) were significantly reduced by 36.61% and 55.52% at Kangping in 2013 and 2014, and significantly reduced by 60.7% at Daqing in 2013 after SN101 treatment (*P* ≤ 0.05), respectively, when compared to the control. We also found the cysts per 100 cm^3^ of soil (Fig 3B) were significantly reduced by 19.62% and 12.97%, respectively, after SN101 treatment when compared to the control (*P* ≤ 0.05) at Kangping in 2013 and 2014, and reduced 37.4% and 21.89%, respectively, at Daqing in 2013 and 2014. After BFA treatment, the cysts per 100 cm^3^ of soil were significantly reduced by 35.53% and 14.77% when compared to the control (*P* ≤ 0.05) at Kangping in 2013 and 2014, and reduced by 28.78% and 16.51% at Daqing in 2013 and 2014 (Fig 3B). However, there were no significant difference in the cyst numbers among SN101, BFA treatment and control (*P* ≤ 0.05) at Daqing in 2014. Moreover, the number of juveniles inside the soybean roots was reduced by 61.8% and 53.51% at Kangping, and reduced by 50.65% and 85.95% at Daqing after SN101 treatment when compared to the uncoated control from 2013 to 2014 (Fig 3C). Furthermore, the egg number per cyst was significantly inhibited after SN101 seed coating or BFA seed coating when compared with the uncoated control (Fig 3D).

**Fig 4:**
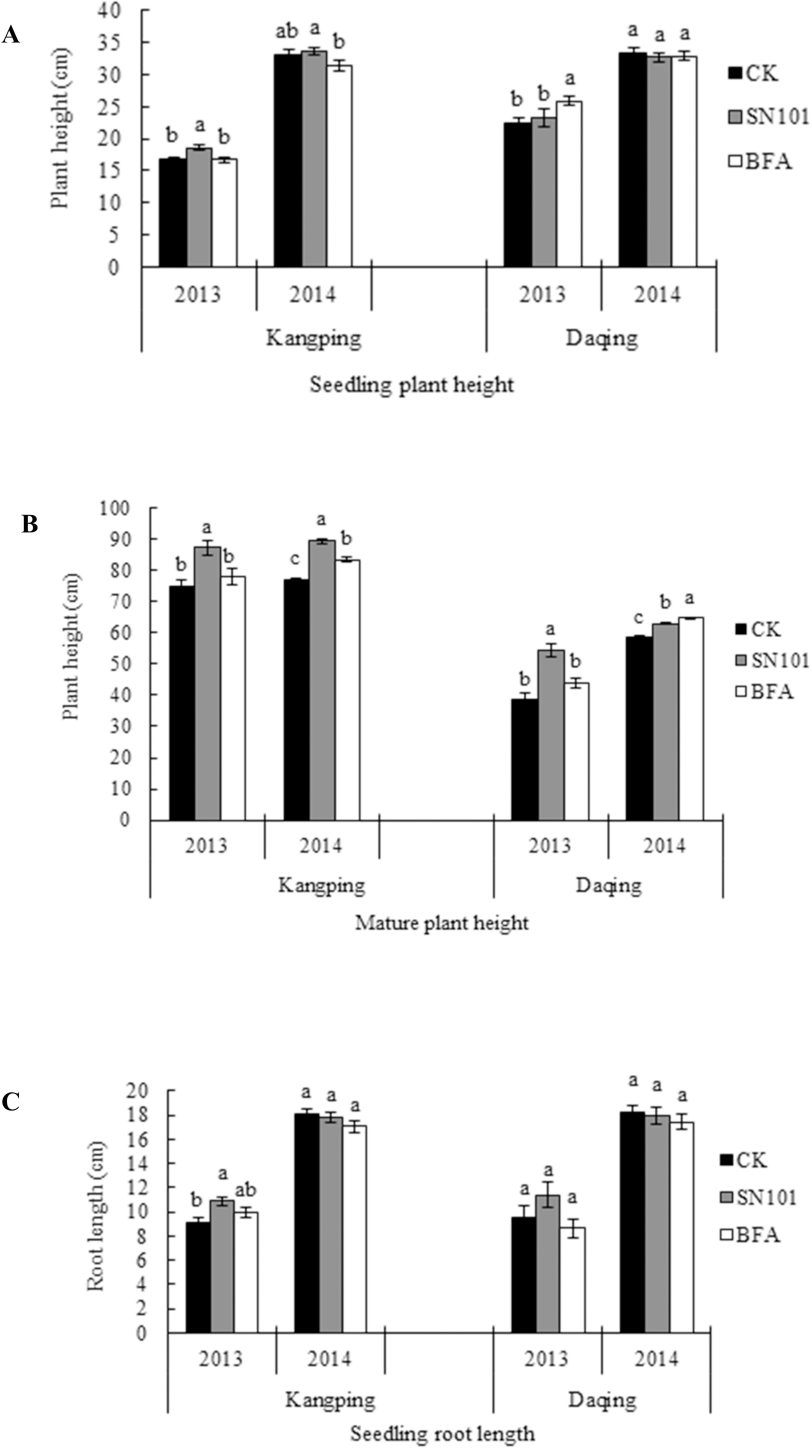
Effects of SN101 seed coating on soybean height and root length under filed conditions in 2013 and 2014. Plants were treated with biocontrol seed coating-sn101, chemical seed coating-BFA and uncoated-CK. The seedling plant height, mature plant height and seedling root length was measured in seedling (after planted 30–35 days) and mature (about after planted 150 days). The data in the figure are mean ± SE and means on the same column followed by different letters indicate significant differences based on a LSD test (*P* ≤ 0.05, n = 125). **A.** seedling plant height, **B.** mature plant height, **C.** seedling root length.

Multiple factor ANOVA analysis showed that years, sites and treatments had a significant effect on the soybean growth except for the effect of sites on the seedling root length and the effect of treatments on seedling plant height. There are limited interactions among the factors: years, sites, and treatments except for significant interactions between years and treatments (Table 3). Although no significant differences were observed in soybean plant height at seedling stage between treatments (Fig 4A), the mature heights were significantly increased after SN101 treatment (*P* ≤ 0.05) when compared to the uncoated control (Fig 4B) except for BFA treatment at Daqing in 2014. On the other hand, we found that the soybean root length at seedling stage was not significant different among these treatments except at Kangping in 2014.

Multiple factor ANOVA analysis suggested that years, sites and treatments had a significant effect on the soybean yield. But there are limited interactions among the factors: years, sites, and treatments except for significant interactions between years and sites (Table 3). The soybean yield was significantly increased by 30.09% and 51.08% (*P* ≤ 0.05), respectively, when applying SN101 seed coating, and increased by 11.15% and 24.63%, respectively, in BFA treatment, when compared to untreated control in the fields of Kangping and Daqing (Table 4). The soybean yield enhancement was due to the significantly increased number of available pods and seeds per plant after SN101 seed coating and BFA treatment compared to untreated control, but the increase rate was significant higher for the SN101 seed coating. There are no significant difference in the weight of 100 seeds in SN101 and BFA treatment compared to untreated control.

**Table 4:** Effect of SN101 on soybean yields under field conditions in Kangping and Daqing in 2013 and 2014 (*P* ≤ 0.05).

		Kangping				Daqing			
		Pods number per plant	Seeds number per plant	100-seed weight (g)	Yield increase rate (%)	Pods number per plant	Seeds number per plant	100-seed weight (g)	Yield increase rate (%)
2013	CK	47.2±2.97a	92.48±6.1a	22.06±.68a	——	22.96±1.43c	61.2±4.1c	14.62±.31a	——
	SN101	51.96±2.40a	103.52±5.25a	23.09±.63a	30.44	40.72±1.4a	89.08±4.93a	16.22±.76a	65.04
	BFA	52.92±2.995a	94.96±6.3528a	23.9±.51a	5.19	30.24±1.97b	69.52±5.83b	14.94±.66a	20.2
2014	CK	31.24±.64c	54.67±1.15c	28.97±.29a	——	34.52±.79b	86.78±2.45b	18.4±.51a	——
	SN101	37.68±.69a	66.72±.94a	29.48±.65a	29.75	37.712±.62a	100.57±1.87a	19.44±.52a	37.12
	BFA	34.06±.69b	62.92±1.29b	29.12±.43a	17.11	36.8±.71a	97.33±2.18a	19.09±.37a	29.06

Plants were treated with biocontrol seed coating-SN101, chemical seed coating-BFA and uncoated-CK. The data in the table are mean ± SE and means on the same column followed by different letters indicate significant differences based on a LSD test (*P* ≤ 0.05, n = 125).

To further validation the effect of SN101 seed coating on soybean production, the final yields of soybeans were measured from 2012 to 2016. The results showed that soybean yields in the two stations were increased by 9.99–25.38% in Heihe Station and by 7.5–16.63% in Jiusan Station, respectively after SN101 seed coating treatment (Table 5).

**Table 5:** Effect of SN101 on final yields of soybeans under field conditions in Heihe from 2012 to 2015 and in Jiusan from 2012 to 2016.

Year	Final yields in Heihe Station (Kg/ha)		Final yields in Jiusan Station (Kg/ha)	
	CK	SN101	CK	SN101
2012	1773	2223	2010	2200
2013	1151	1266	2262	2475
2014	2140	2446	3000	3225
2015	1698	1961	3000	3499
2016	1247	1543	1395	1562

Plants were treated with biocontrol seed coating-SN101 and uncoated-CK.

## Discussion

We, for the first time, examined the effect of co-applying three rhizobacterial strains on nematode control under greenhouse and field conditions in China. Our results suggest that SN101 is a promising biocontrol agent for managing SCN. The role of SN101 in biological control of nematodes is very stable in the two field sites (Kangping and Daqing) for two years, and two stations (Heihe and Jiusan) for five yearsindicating it can be widely used in the fields. Compared to multiple applications of chemical nematicides in one growth season, one application of seed-coating SN101 will reduce dosage of chemical nematicide application, which considerably reduces the environmental pollution.

The combination of the three SN101 strains could inhibit SCN reproduction. Previous studies showed that combination of *Monacrosporium lysipagum* and *Paecilomyces lilacinus* reduced population of *Radopholussimilis*, *Heterodera avenae* and *Meloidogyne javanica* in banana, barley plant and tomato, respectively [28], indicating that combination of various biocontrol agents provides enhanced control effect over a single agent. Taking the nematode lethality rate and soybean yield increase rate into consideration, the current study has provided evidence that *B*. *simple*, *B*. *megaterium* and *S*. *fredii* applied in 3:1:1 ratio is suitable for controlling nematodes on soybean under natural field conditions. The three strains did not show any antagonism among them. Previous study showed that each of *B*. *simple*, *B*. *megaterium* and *S*. *fredii* for nematode control efficacy (Cyst number per root) respectively reached 47.92%, 41.8% and 46.2% in the field conditions in Daqing, Anda and Kangping in 2011 (S1 Table). Our result suggested that combination of these three biocontrol strains provides enhanced stably control effect over a single strain. Most rhizobacteria reduced nematode populations by metabolic products, such as toxins and enzymes [29]. However, how SN101 mechanisms as the effective biological control agent remains are unknown in here. There may be synergistic effect among three bacteria strains, which enhances the effect of nematode control. So in the next step, we are exploring the biochemistry and molecular mechanisms of SN101 in nematode control. Perhaps one of mechanisms is that co-application of various bacteria may augment overlay the generation of toxins, enzymes or metabolites from bacteria and plants.

In summary, application of soybean seed-coating with different biocontrol strains not only has lethal effects on nematodes but also enhances the yields of soybean plants. Such effect is better than commercial seed coating agent. The results obtained in this study are highly encouraging and suggests that seed treatment with SN101 may represent a safe economic and effective alternative way to use nematicides in commercial soybean cultivation.

## Supporting information

S1 Table Effect of the three strains on the number of cysts. Plants were treated with *Bacillus simple* (Sneb545), *B*. *megaterium* (Sneb482) and *Sinarhizobium fredii* (Sneb183) under three field conditions in Daqing City of Heilongjiang Province, Anda County of Heilongjiang Province and Kangping County of Liaoning Province, respectively in 2011. The data in the table are mean ± SE and means on the same column followed by different letters indicate significant differences based on a LSD test (*P* ≤ 0.05, n = 125).
(DOCX)
