# Correction: Management of the soybean cyst nematode *Heterodera glycines* with combinations of different rhizobacterial strains on soybean

**DOI:** 10.1371/journal.pone.0194287

**Published:** 2018-03-08

**Authors:** Yuanyuan Zhou, Yuanyuan Wang, Xiaofeng Zhu, Rui Liu, Peng Xiang, Jingsheng Chen, Xiaoyu Liu, Yuxi Duan, Lijie Chen

Fig 1 is a duplicate of Fig 2. Please see the correct Fig 1 here.

**Fig 1:**
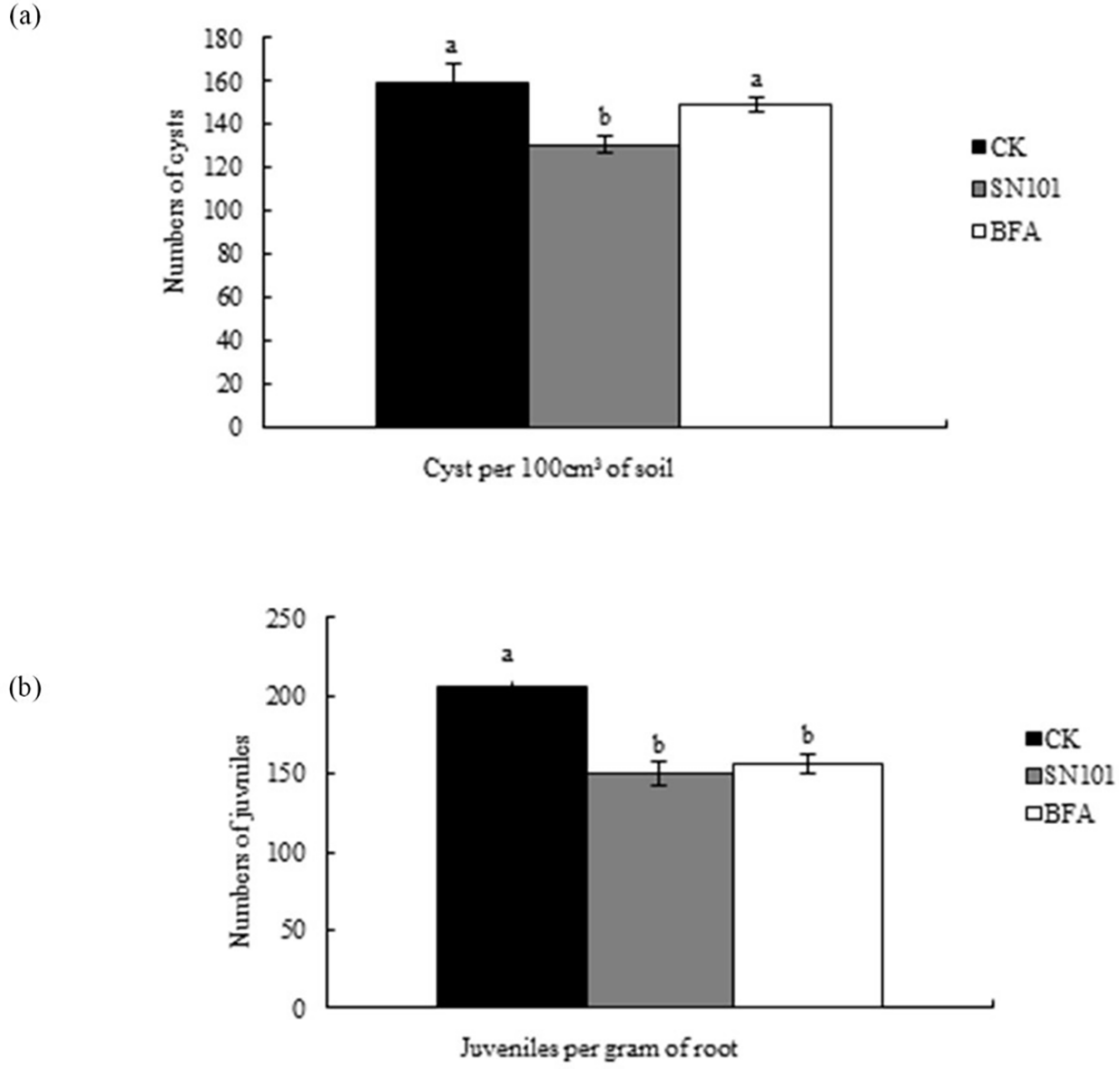
Effects of SN101 seed coating on the control of SCN infestation in soybean under greenhouse condition. Plants were treated with biocontrol seed coating-SN101, chemical seed coating-BFA and uncoated-CK. The number of juveniles and cysts was measured after nematode inoculation 30 days. The data in the figure are mean ± SE and means on the same column followed by different letters indicate significant differences based on a LSD test (P ≤ 0.05, n = 12).
