# Supplementary material for: Management of the soybean cyst nematode Heterodera glycines with combinations of different rhizobacterial strains on soybean
Source: PLoS One. 2017 Aug 3;12(8):e0182654. doi: 10.1371/journal.pone.0182654 (PMC5542665; doi:10.1371/journal.pone.0182654)
Supplement: S1 Table — Plants were treated with Bacillus simple (Sneb545), B. megaterium (Sneb482) and Sinarhizobium fredii (Sneb183) under three field conditions in Daqing City of Heilongjiang Province, Anda County of Heilongjiang Province and Kangping County of Liaoning Province, respectively in 2011. The data in the table are mean ± SE and means on the same column followed by different letters indicate significant differences based on a LSD test (P ≤ 0.05, n = 125). (DOCX) [file pone.0182654.s001.docx]

S1 Table. Effect of the three strains on the number of cysts.

| Treatment | The number of cyst per root |
| --- | --- |
| CK (Daqing) | 87.08±8.12a |
| Sneb545 (Daqing) | 45.35±4.73b |
| CK (Anda) | 27.5±4.32a |
| Sneb482 (Anda) | 16±2.12b |
| CK (Kangping) | 117±9.3a |
| Sneb183 (Kangping) | 63±7.14b |

Plants were treated with *Bacillus simple* (Sneb545), *B. megaterium* (Sneb482) and *Sinarhizobium fredii* (Sneb183) under three field conditions in Daqing City of Heilongjiang Province, Anda County of Heilongjiang Province and Kangping County of Liaoning Province, respectively in 2011. The data in the table are mean ± SE and means on the same column followed by different letters indicate significant differences based on a LSD test (*P* ≤ 0.05, n=125).
